# Chitinase-Like Protein PpCTL1 Contributes to Maintaining Fruit Firmness by Affecting Cellulose Biosynthesis during Peach Development

**DOI:** 10.3390/foods12132503

**Published:** 2023-06-27

**Authors:** Ze Xu, Jieyu Dai, Liping Liang, Yonglan Zhang, Yaojun He, Libo Xing, Juanjuan Ma, Dong Zhang, Caiping Zhao

**Affiliations:** College of Horticulture, Northwest Agriculture and Forestry University, Yangling, Xianyang 712100, China; xuzeizi@163.com (Z.X.);

**Keywords:** cellulose, chitinase-like protein, firmness, peach

## Abstract

The firmness of the flesh fruit is a very important feature in the eating process. Peach fruit is very hard during development, but its firmness slightly decreases in the later stages of development. While there has been extensive research on changes in cell wall polysaccharides during fruit ripening, little is known about the changes that occur during growth and development. In this study, we investigated the modifications in cell wall components throughout the development and ripening of peach fruit, as well as its impact on firmness. Our findings revealed a significant positive correlation between fruit firmness and cellulose content at development stage. However, the correlation was lost during the softening process, suggesting that cellulose might be responsible for the fruit firmness during development. Members of the chitinase-like protein (CTL) group are of interest because of their possible role in plant cell wall biosynthesis. Here, two CTL homologous genes, *PpCTL1* and *PpCTL2*, were identified in peach. Spatial and temporal expression patterns of *PpCTLs* revealed that *PpCTL1* exhibited high expression abundance in the fruit and followed a similar trend to cellulose during fruit growth. Furthermore, silencing *PpCTL1* expression resulted in reduced cellulose content at 5 DAI (days after injection), this change that would have a negative effect on fruit firmness. Our results indicate that *PpCTL1* plays an important role in cellulose biosynthesis and the maintenance of peach firmness during development.

## 1. Introduction

Peaches (*Prunus persica* L. Batsch) are a popular commercial crop in the world, cherished by consumers for their distinctive flavor. However, after harvest, peaches experience a rapid decline in firmness, leading to a short storage period and limitations in transportation distance and economic value [1]. Improving fruit firmness while maintaining fruit quality is a primary objective in modern peach breeding. Consequently, understanding the mechanism underlying fruit firmness holds immense significance for improving peach commodity.

During fruit development and ripening, fruit firmness undergoes significant changes [2]. It depends on the structure of the cell wall polysaccharides and intracellular expansion pressure [3]. The plant cell wall is composed of polysaccharides (cellulose, hemicellulose and pectin) and structural proteins. [4]. The dynamic changes of cell wall polysaccharides alter fruit firmness through the involvement of various cell wall hydrolases [5,6]. Polygalacturonase (PG), pectin lyases (PL) and pectin methylesterase (PME) play a crucial role in pectin depolymerization [2]. Inhibiting the expression of cell wall modification genes can help maintain fruit firmness and prevent softening. For example, in peaches, the inhibition of pectin lyase (*PpePL1* and *PpePL15*) and polygalacturonase (*PpPG21* and *PpPG22*) gene expression levels prevented pectin depolymerization and delayed fruit softening [7,8]. While the changes in fruit firmness have been well documented at the storage stage, there is limited information on changes during fruit development [9]. Brummell et al. [6] discovered that matrix glycans in the cell wall began to depolymerize before peach ripening, indicating that cell wall components during development also play a role in fruit softening. Furthermore, pre-harvest fruit quality is a prerequisite for post-harvest fruit quality. Thus, it is important to understand the dynamic changes in peach fruit firmness during development and maturation.

Cellulose is composed of β-D-(1-4) glucose chains. The glucose chain can be linked by hydrogen bonds to form crystalline microfibrils, which further form the higher structure of cellulose and become the basic skeleton of the cell wall [10]. Cellulose plays an important role in various aspects of plant growth, such as root hair elongation, pollen tube germination, plant stature, stem strength and response to biotic and abiotic stresses [11,12,13,14,15]. Additionally, studies have shown its effect on fruit firmness. In persimmon, strawberry and apple, the softening process is accompanied by a continuous decrease in cellulose content [16,17,18]. In avocados, the loss of cohesion within the fiber structure and the altered binding of cell wall matrix polysaccharides with the onset of fruit softening suggest that cellulose depolymerization leads to loss of pericarp firmness [19]. Conversely, research on tomato and mango has shown that cellulose content remains relatively stable during softening [20,21]. These results suggest that the involvement of cellulose in fruit softening is different among various species. Consequently, further investigations are needed to explore the correlation between cellulose and fruit firmness in peaches.

Chitinase (EC 3.2.1.14), a subgroup of glycosyl hydrolases (GH), catalyzes the hydrolysis of chitin by cleaving the β-1,4 linkage of N-acetylglucosamine. Chitinases are divided into five classes (class I to V) based on domain and motif structure [22]. The Class I, II and IV chitinases belonging to GH18 are widely present in various organisms, including humans, fungi, bacteria, yeasts, plants and animals, while the Class III and V chitinases belonging to GH19 are only exclusively in high plants [22,23]. Since the plant cell wall does not contain chitin, when chitin-containing pathogens infect plants, the expression of chitinase genes is activated to induce PAMP (pathogen-associated molecular pattern)-triggered immunity; therefore, chitinase is considered to be a pathogenesis-related (PR) protein [24]. Several lines of evidence suggest that transgenic expression of chitinases increases resistance to fungal pathogens [23,25]. In addition, many chitinases exhibit tissue- and development-specific expression patterns in plants, playing important roles in the growth and development processes such as pollination, senescence, root development, seed germination and somatic embryogenesis [26,27,28,29]. The absence of the secretory chitinase *EP3* in the *ts11* carrot cell mutant leads to abnormal embryonic development, which can be rescued by adding chitinase [30]. Additionally, a T-DNA insertion mutant of the chitinase protein *OsCLP* in rice causes significant growth retardation, including dwarfism, reduced primary root length and an effect on cortical cell size in the elongation zone [26]. Overexpression of *OsCLP* alters seed shape and increases root and shoot length as well as leaf size [26]. In bananas, Class III chitinase is abundant in unripe pulp and acts as a fruit-specific vegetative storage protein, providing a source of amino acids for the synthesis of ripening-associated proteins [31]. These findings indicate that chitinase serves a non-defensive function in plant growth and development.

Among that, the function of Class II chitinase-like proteins (CTL) has been extensively investigated. In *Arabidopsis*, *AtCTL1* and *AtCTL2*, classified as Class II, are constitutively expressed in multiple organs [27,32]. A mutation in *AtCTL1* causes ectopic lignin deposition, incomplete cell walls, reduced root and hypocotyl length and increased root hair number, disrupting normal plant growth and development [33,34]. In addition, *AtCTL1* and its homolog *AtCTL2* are found to be functionally equivalent, impacting cellulose biosynthesis and playing an important role in determining cell wall rigidity [35]. In maize, the overexpression of the chitinase-like protein1, *ZmCtl1*, enhances the mechanical stalk strength of the stem by increasing cellulose content, ferulic acid and Ara sugar [13]. Moreover, RNA-seq analyses reveal that CTL group genes are highly coordinated with the expression of cellulose synthase genes in cotton and flax [13,36]. These results indicate that the functions of *CTLs* are important for cellulose synthesis. In the present study, we revealed the significant differences in endocarp cell wall composition during peach fruit development and ripening. We also identified chitinase-like proteins in peaches and further elucidated the function of *PpCTL1* in cellulose biosynthesis and fruit firmness. Therefore, our research provides new insights into the mechanism of maintaining peach fruit firmness and lays the foundation for further studies on the function of chitinase in peaches.

## 2. Materials and Methods

### 2.1. Plant Materials

Peach trees (*Prunus persica* L. Batsch, cv ‘Shuang Xi Hong’) were grown at the Experimental Station of the College of Horticulture at Northwest A & F University, Yangling, Shaanxi, China. Tissues from roots (1–2 mm diameter), stems (fresh shoot), mature leaves, flowers (fully blooming) and young fruit (30 days after blooming, DAB) were collected to analyze tissue-specific gene expression. Developmental fruit were sampled at 20 DAB (Stage1, S1), 35 DAB (S2), 50 DAB (S3) and 65 DAB (S4). At each developmental stage, a minimum of 20 fruits were collected and weighed. At the commercial mature period (75 DAB, fruit was partially colored, firmness about 60 N), the fruit was harvested and stored in a storage room at a temperature of 25 ± 1 °C and a relative humidity of 75–85%. During storage, flesh samples were taken at 0 d, 2 d, 4 d, 6 d and 8 d. Three biological replicates were set up, and each replicate consisted of 6 fruits.

### 2.2. Fruit Firmness Determination

Fruit firmness was measured using a GY-4 penetrometer (Top Instrument Co., Hangzhou, China) equipped with a 7.9 mm cylindrical probe. After peeling the fruit, two symmetrical positions on the equatorial plane were selected for firmness measurement [8].

### 2.3. Measurement of Pectin and Cellulose Content

The determination of pectin content followed the method described by He et al. [37]. Approximately 1 g of peach flesh was taken, crushed and boiled with 10 mL of 95% ethanol (*v*/*v*) for 10 min. The boiling process was repeated multiple times to eliminate sugars and neutral sugars. After cooling, the supernatant was removed by centrifugation. The collected precipitate was suspended in 10 mL of doubly distilled water (ddH_2_O) and heated at 50 °C for 30 min. The supernatant and precipitate were then collected separately. The supernatant was utilized to determine the water-soluble pectin (WSP), while the precipitate was suspended in 0.5 M H_2_SO_4_, boiled in a water bath for 1 h and subsequently centrifuged. The resulting supernatant was used for the determination of acid-soluble pectin (ASP). A carbazole—H_2_SO_4_ colorimetric method was used for the determination of WSP and ASP. The cellulose content was measured using a cellulose assay kit (Solarbio, BC4280, Beijing, China) according to the manufacturer’s protocol. Three biological replicates were designed, and each replicate was composed of at least 6 fruits.

### 2.4. PpCTL Protein Identification, Sequence Alignment and Phylogenetic Tree Construction

The protein sequences of AtCTL1 (AT1G05850.1) and AtCTL2 (AT3G16920.1) were downloaded from the Arabidopsis information resource database (http://www.arabidopsis.org/index.jsp, (accessed on 12 May 2022). To identify the chitinase-like (CTL) proteins in peach, the protein sequences of AtCTL1 and AtCTL2 were used as queries to search the peach genome database (*Prunus persica* genome v2.0.a1). All candidate gene sequences were manually checked using the Pfam website (https://www.ebi.ac.uk/interpro, accessed on 15 June 2022) to confirm the presence of conserved domains. Multiple amino acid sequence alignments were performed by DNAMAN 6.0 software. Phylogenetic trees, including PpCTLs and other known CTL members, were constructed using MEGA 6.0 software with the neighbor-joining (NJ) method. Bootstrap was set to 1000 for iterative boot testing [38].

### 2.5. Total RNA Extraction and Gene Expression Analysis

Total RNA was extracted from peach flesh and various tissues using the CTAB method as described by Qian et al. [7]. The extracted RNA was subjected to 1.5% agarose gel electrophoresis, and its concentration was determined using a UV spectrophotometer. According to the manufacturer’s protocol, 1 μg of RNA was reverse transcribed into first-strand cDNA using the PrimeScript RT Reagent Kit with gDNA Eraser (TaKaRa, Dalian, China). Real-time quantitative PCR (RT-qPCR) was conducted using the Bio-Rad CFX system (Bio-Rad, Hercules, CA, USA). Each reaction was carried out with a volume of 10 µL and quantified in triplicate. The reaction contains 10 ng/µL cDNA, 1 µL of forward and reverse primer and 5 µL of 2× SYBR Premix Ex Taq II (TaKaRa, Dalian, China). Some ddH_2_O is added to 10 µL. *PpCYP2* (*Prupe.8G233900*) and *PpTua5* (*Prupe.6G004100*) were used as the internal controls [39]. The melting curve and no-template control (NTC) were used to confirm the specificity of the reaction. The relative expression level was calculated by the 2^−ΔΔCt^ method [40]. Primers used for RT-qPCR are listed in Table S1 (Supplementary Materials).

### 2.6. Virus-Induced Gene Silencing (VIGS)

The specific C-terminal fragment of *PpCTL1* was amplified into the pTRV2 plasmid to form a gene silencing plasmid (pTRV2-*PpCTL1*). The primers used for amplification are provided in Table S1 (Supplementary Materials). The VIGS assay followed the method described by Xu et al. [8]. At 60 DAB, fruit were injected with a suspension containing *Agrobacterium* harboring *pTRV2* and *pTRV1* for control fruit and *pTRV2*-PpCTL1 and *pTRV1* for RNAi fruit. Each fruit was injected symmetrically with 500 μL of the suspension. The control fruit and RNAi fruit were harvested at 5, 10 and 15 days after injection (DAI) and transported to the laboratory. The injection fruit flesh was used for firmness determination, gene expression analysis, cell wall composition measurement and paraffin section assay. Three biological replicates were designed, and each replicate was composed of at least 6 fruits.

### 2.7. Paraffin Section Assays

Flesh tissue blocks of 3–5 cm^3^ were placed in Formalin-Aceto-Alcohol (FAA) solution for 48 h, then removed and rinsed with ddH_2_O. The flesh tissue was then dehydrated through a gradient of ethanol (30%, 50%, 70%, 85%, 95% and 100%), transparentized through a gradient of 100% ethanol to 100% xylene and embedded in paraffin. Sections were sliced with a paraffin slicer and adhered to slides [7]. All sections were rehydrated and stained with toluidine blue and Safranin O-Fast Green stain. Sections were photographed under the microscope (BX51, Olympus, Japan), and this was repeated three times to obtain approximately nine photographs of each sample. Cell walls containing cellulose appeared green under Safranin O-Fast Green staining and blue-purple under toluidine blue staining. 

### 2.8. Statistical Analysis

Means and standard deviations (SDs) were calculated using Microsoft Excel software version 2019. The experiments were arranged in a completely randomized design. For each measurement, at least three biological replicates were conducted. Additionally, each replicate was composed of at least 6 fruits. SPSS Statistics software version 23.0 was used to analyze the homogeneity of variance and normal distribution of all original data, followed by analysis of significant differences. Since the raw data of *PpCTL2* expression during development conformed to normal distribution but not homogeneity of variance, the non-parametric variance analysis test was used to analyze the significant differences of *PpCTL2* expression levels at different developmental time points. Multi-sample significance analysis was used to analyze the sample data of fruit firmness, pectin, cellulose content and *PpCTL1* gene expression level at different fruit development stages. Multiple-sample significance test was calculated with a one-way ANOVA followed by Duncan’s (*p* < 0.05). Two-sample significance analysis was used to analyze the sample data from control and RNAi fruit at same time points. Two-sample significance test was performed with Student’s *t*-test (*, *p* < 0.05). Correlation analysis was performed by SPSS software version 23.0. The figure was prepared using Origin 2018.

## 3. Results

### 3.1. Changes in Fruit Firmness and Cell Wall Composition during Peach Fruit Development and Ripening

**Figure 1:**
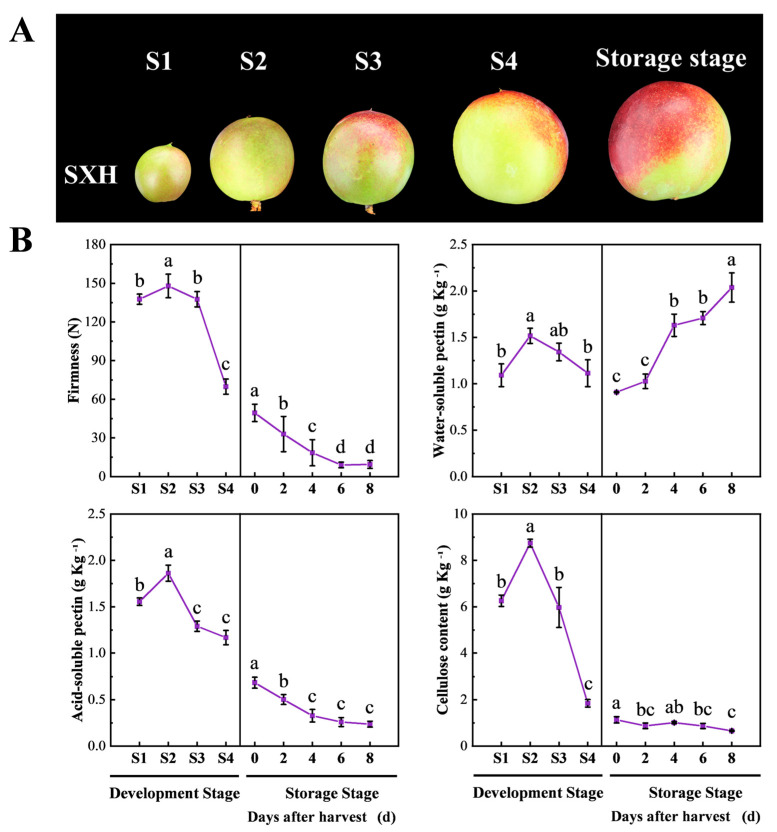
Changes in firmness, cell wall composition and PG activity during peach fruit development and ripening. (**A**) Fruit status at different stages: S1 (20 DAB, days after blooming), S2 (35 DAB), S3 (50 DAB), S4 (65 DAB) and storage stage (75 DAB). Scale bars = 1 cm. (**B**) Change in fruit firmness, WSP (water-soluble pectin), ASP (acid-soluble pectin) and cellulose at different stages. At 75 DAB, the fruits were harvested and stored in a storage room. Flesh samples were collected at 0, 2, 4, 6 and 8 days after harvest. Data are expressed as means ± SD from at least three samples. Different lowercase letters in the figure denote significant differences between sampling dates for fruit by Duncan’s multiple range test (*p* < 0.05).

Peach fruit growth can be distinguished into four stages (S1–S4). The first stage (S1) is characterized by a rapid increase in fruit volume and weight, accompanied by rapid fruit expansion. During the second stage (S2), fruit growth slows down, and the endocarp gradually undergoes lignification. In the third stage (S3), the fruit experiences another significant increase in size, reaching up to three times its initial weight in less than 20 days. In the fourth stage (S4), the fruit reaches its full size and subsequently begins to ripen and soften (Figure 1A, Supplementary Materials, Figure S1). In order to explore the changes in fruit firmness and cell wall components throughout development and ripening, four developmental stages (S1, S2, S3, S4) and five time points during softening (0, 2, 4, 6, 8 days after harvest) were selected (Figure 1B). During the developmental stage, fruit firmness remained high from S1 to S3 and then rapidly decreased from S3 to S4 stage. During storage, fruit firmness continued to decrease. The changes in acid-soluble pectin (ASP) content were consistent with the variations in firmness during fruit development and softening. Water-soluble pectin (WSP) content exhibited a similar trend to firmness during fruit development, while gradually increased after harvest, displaying an opposite pattern compared to firmness. The cellulose content obviously was enhanced from S1 to S2, followed by a sharp decline from S2 to S4, and remained at a low level during storage.

### 3.2. Correlation between Fruit Firmness and Cell Wall Composition at Different Developmental Stages

Considering the similar trends observed in the changes of acid-soluble pectin and cellulose content alongside firmness during fruit development and softening, we investigated the correlations between fruit firmness and the levels of pectin and cellulose at different stages (Table 1). Correlation analysis revealed a positive relationship between fruit firmness and cellulose content during the developmental stage. However, during the storage stage, fruit firmness showed a positive correlation with acid-soluble pectin (ASP) but a negative correlation with water-soluble pectin (WSP). These findings provide evidence that cellulose may serve as the fundamental framework of the cell wall, maintaining the firmness of peach fruit during development. Conversely, the decline in firmness during softening is associated with the degradation of ASP and the increase in WSP content.

### 3.3. Sequence Analysis of PpCTL1 and PpCTL2

**Figure 2:**
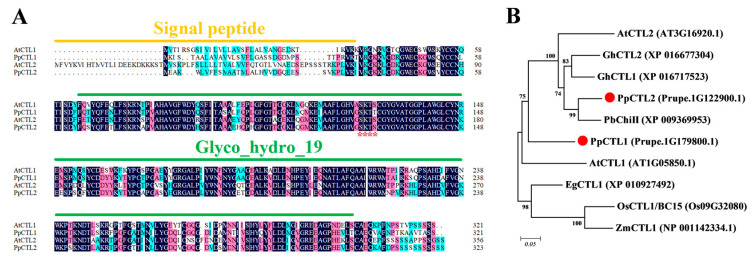
Sequence analysis of PpCTL1 and PpCTL2. (**A**) Multiple sequence alignment of PpCTL1/2 and AtCTL1/2 proteins. Different colors indicate different similarities (black: 100%, magenta: 75% and blue: 50%). Glyco_hydro_19 represents the conserved domain of the glycosyl hydrolases 19 family. The signal peptides were analyzed by Signal 4.0. The red star indicates the amino acid residue S-K-T-S lacking catalytic activity. Multiple amino acid sequence alignments were performed by DNAMAN 6.0 software. (**B**) Phylogenetic analysis of PpCTL1/2 and partial CTL proteins from *Arabidopsis thaliana*, cotton (*Gossypium hirsutum*), pear (*Pyrus x bretschneideri*), rice (*Oryza sativa*), oil palm (*Elaeis guineensis*) and maize (*Zea mays*). Phylogenetic trees were constructed using MEGA 6.0 software with the neighbor-joining (NJ) method.

As chitinase-like (CTL) protein has been reported to be involved in cellulose synthesis and to play a key role in establishing interactions between cellulose microfibrils and hemicellulose, we focused on investigating the function of CTL in peaches. In this study, two CTL homologous genes (Prupe.1G122900.1 and Prupe.1G179800.1) were identified. Multiple sequence comparisons revealed that both Prupe.1G122900.1 and Prupe.1G179800.1 contain a Glyco_hydo_19 conserved domain, indicating that they belong to the glycosyl hydrolase 19 family (Figure 2A). Based on the phylogenetic tree, Prupe.1G179800.1 exhibited high homology with AtCTL1 and was therefore named PpCTL1, while Prupe.1G122900.1 was named PpCTL2 (Figure 2B). The PpCTL1 peptide shares 73.68% sequence identity with AtCTL1 proteins, while the PpCTL2 peptide shares 71.43% sequence identity with AtCTL2 proteins. PpCTL1/2 differs from AtCTL1/2 in the N-terminal signal peptide, and Pfam analysis showed that they belong to class II chitinase. Furthermore, the glyco_hydro_19 domain of PpCTL1 and PpCTL2 contains an S-K-T-S amino acid residue, replacing the H-E-T-T motif, which is essential for chitinase activity. This suggests that PpCTL1 and PpCTL2 may not possess chitinase activity (Figure 2A).

### 3.4. PpCTL1 Expression Is Closely Associated with Cellulose Content and Firmness

**Figure 3:**
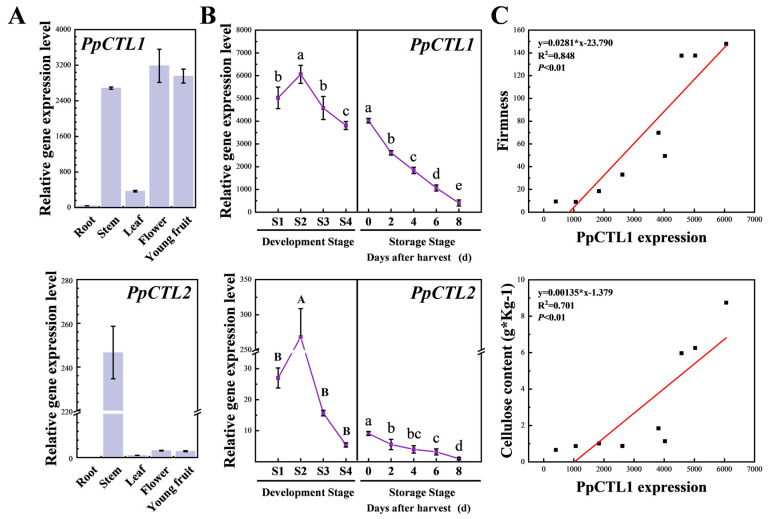
The gene expression level of *PpCTL1* and *PpCTL2* in peach. (**A**) Relative expression levels of *PpCTL1* and *PpCTL2* in different tissues. (**B**) Relative expression levels of *PpCTL1* and *PpCTL2* at various developmental stages of fruit. Data are expressed as means ± SD from at least three samples. Different lowercase letters in the figure denote significant differences between sampling dates for fruit by Duncan’s multiple range test (*p* < 0.05). Different uppercase letters in the figure indicate significant differences by non-parametric variance analysis test. (**C**) Correlation analysis between the expression levels of *PpCTL1* with firmness and cellulose content at different developmental stages. P is correlation coefficient. Each RT-qPCR was normalized using the cycle threshold value of the *PpCYP2* (Prupe.8G233900) and *PpTua5* (Prupe.6G004100). The relative expression level was calculated by the 2^−ΔΔCt^ method.

To investigate the spatial and temporal expression patterns of *PpCTLs*, RT-qPCR was used to detect the expression level of *PpCTL1* and *PpCTL2* in various tissues and at different developmental stages (Figure 3). The expression level of *PpCTL1* was higher in the stem, flower and young fruit compared to the root and leaf. *PpCTL2* exhibited high expression only in the stem. During the fruit development stage, the expression level of *PpCTL1* and *PpCTL2* increased from S1 to S2 and subsequently decreased significantly from S2 to S4. At the storage stage, the expression of *PpCTL1* and *PpCTL2* continuously decreased. In addition, the transcript abundance of *PpCTL1* in fruit was significantly higher than that of *PpCTL2*. Therefore, we further evaluated the correlations between *PpCTL1* expression with fruit firmness and cellulose content at different stages. Linear regression analysis showed that the *PpCTL1* expression was correlated with cellulose content (R^2^ = 0.701, *p* < 0.01) and firmness (R^2^ = 0.848, *p* < 0.01). Consequently, *PpCTL1* likely plays an important role in the cellulose accumulation of peach fruit, based on its high expression level, spatial and temporal expression pattern and correlation with cellulose content.

### 3.5. Downregulated PpCTL1 Expression Affects Fruit Firmness

**Figure 4:**
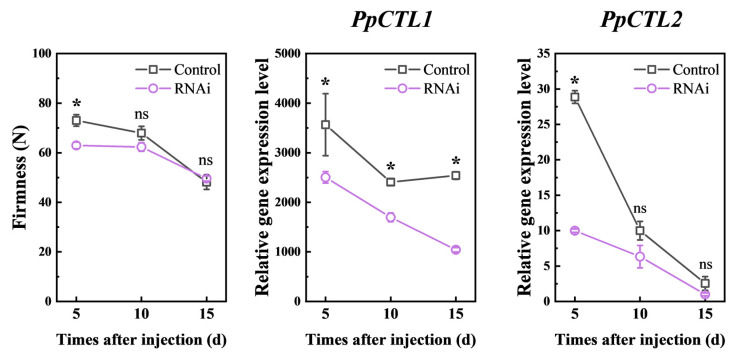
Changes in the firmness and relative expression of *PpCTL1* and *PpCTL2* of control (TRV2) and RNAi (TRV2-PpCTL1) fruit. Data are expressed as means ± SD from at least three samples. The asterisk indicated a significant difference (*p* < 0.05), using the two-tailed Student’s *t*-test. ns, no significant. Each RT-qPCR was normalized using the cycle threshold value of the *PpCYP2* (Prupe.8G233900) and *PpTua5* (Prupe.6G004100). The relative expression level was calculated by the 2^−ΔΔCt^ method.

In order to further investigate the function of *PpCTL1* in peach fruit development and ripening, a virus-induced gene silencing vector targeting the C-terminal specific fragment of *PpCTL1* was generated (Supplementary Materials, Figure S2). Fruit at stage S4 (60 DAB) were injected with *Agrobacterium* harboring the empty vector and the RNAi vector. RT-qPCR analysis showed that the mRNA levels of *PpCTL1* in RNAi fruit were significantly lower than those in control fruit after injection (Figure 4). The similarity between the protein sequences of *PpCTL1* and *PpCTL2* was 71.93%, and the similarity between their coding DNA sequences (CDS) was 70.05% (Supplementary Materials, Figure S3). Therefore, we also investigated the effect of the downregulation of *PpCTL1* expression on PpCTL2 transcript level. The result displayed that *PpCTL2* expression in the RNAi fruit was also inhibited at 5 days after injection (DAI). The firmness of RNAi fruit was significantly lower than that of the control fruit in the first 5 DAI, suggesting that downregulated *PpCTL1* expression affects fruit firmness.

### 3.6. PpCTL1 Impacts Cellulose Content in Fruit

**Figure 5:**
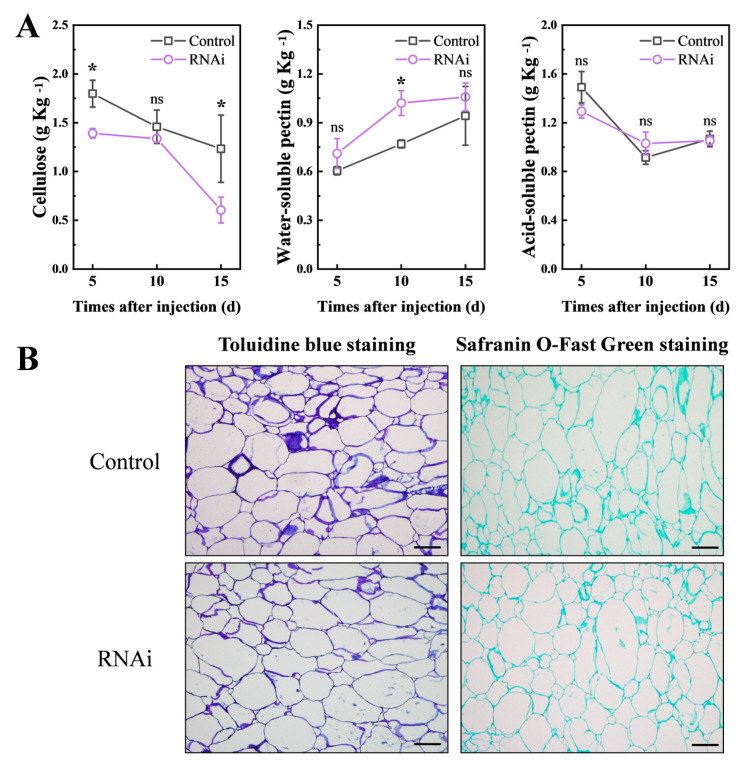
Change in contents of cellulose, water-soluble pectin (WSP), acid-soluble pectin (ASP) and cell wall structure. (**A**) Changes in the cellulose, WSP and ASP of control and RNAi fruit. Data are expressed as means ± SD from three samples. The asterisk indicated a significant difference (*p* < 0.05), using the two-tailed Student’s *t*-test. ns, no significant. (**B**) Cell wall structure was observed by paraffin section. The control and RNAi fruit were collected at 5 days after injection. The sections were stained with toluidine blue and Safranin O-Fast Green staining. Cell walls containing cellulose appeared green under Safranin O-Fast Green staining and blue-purple under toluidine blue staining. Scale bars = 100 μM.

To verify the effect of *PpCTL1* on cellulose synthesis in peach fruit, we examined the cellulose content of RNAi and control fruit (Figure 5). The results showed that, except at 10 DAI, the cellulose content in RNAi fruit was significantly lower than in control fruit. We also assessed the content of pectin substances and found no difference in ASP content between the RNAi and control fruit. However, WSP content was higher in the PpCTL1 down-regulated fruit, and the difference was significant at 10 DAI. Fruit firmness depends on the mechanical strength of the cell wall and cell integrity. To further investigate the cellular structure of RNAi in control fruit, the fruit sections were stained with toluidine blue and fast green FCF, which dyed the cell wall blue and green, respectively. As shown in Figure 5B, the cellular structure of the RNAi fruit remained compact and intact, with no significant differences from the control fruit at 5 DAI. These findings revealed that the decrease in fruit firmness after *PpCTL1* silencing was attributed to a reduction of cellulose content.

## 4. Discussion

### 4.1. Cellulose May Be Responsible for the Peach Fruit Firmness during Development

Fleshy fruit such as tomatoes, apples and peaches undergo a series of developmental steps, including fruit set, fruit growth, maturation and ripening. Different from tomatoes and apples, peaches show a double sigmoidal curve, with two rapid growth stages alternating with two slow growth stages [41]. Peach fruit growth was categorized into four stages (S1–S4) based on its growth curve (Supplementary Materials, Figure S1). During the S1 and S3 stages, the fruit expanded rapidly, while its growth slowed down during the S2 and S4 stages (Figure 1A). In this study, we observed significant changes in the content of cell wall components during fruit development (Figure 1). In *Prunus*, both cytoarchitecture and cell size measurements confirm that cell division is only responsible for the early stages of fruit development, while subsequent growth is fully supported by cell enlargement [42]. During fruit development, which is closely associated with mesocarp cell enlargement, the surface area of the cell wall increases dramatically [42]. Therefore, in addition to intercellular space enlargement, primary cell walls must be rapidly synthesized to increase the wall elasticity required for cell enlargement, resulting in an increase in cellulose and pectin (ASP and WSP) content during S2 (Figure 1B).

The fruit exhibits high firmness during development, with a slight decrease in the late stages of development [6]. Our results also demonstrated that the fruit maintained high firmness from S1 to S3, followed by a significant decrease in firmness at S4. The loss in fruit firmness during development has been confirmed in a variety of fruit, including apples, kiwifruit, strawberries, tomatoes, and mangos [2,18,20]. During development, WSP and ASP showed the same change trend and decreased simultaneously in the late developmental stages (Figure 1B), indicating that ASP did not depolymerize into WSP. Therefore, we hypothesize that the changes in firmness during development are independent of pectin solubilization. Furthermore, we found a significant positive correlation between firmness and cellulose content during development, suggesting that cellulose may influence the firmness of peach fruit during development (Table 1). Similar results have been reported in apples, where cellulose showed a significant correlation with firmness [18]. However, the correlation between cellulose and fruit firmness was lost during peach softening (Table 1). These results suggest that cellulose may contribute to the fruit firmness during the development stage.

### 4.2. Pectin Depolymerization Plays a Central Role in Peach Fruit Softening

After harvested, ripe fruit undergoes a dramatic softening process [5]. Softening is a biological process of primary cell wall remodeling, including pectin depolymerization, hemicellulose solubilization, middle layer dissolution and reduced cell adhesion [2]. Among these changes, pectin depolymerization plays a central role in peach fruit softening [1,7]. Consistent with these findings, our results demonstrated a rapid increase in water-soluble pectin (WSP) content and a decrease in acid-soluble pectin (ASP) content during the storage period, indicating the conversion of insoluble pectin to its soluble form in the middle lamella (Figure 1B). Moreover, fruit firmness exhibited a negative correlation with WSP content and a positive correlation with ASP content (Table 1). There was also a slight decrease in cellulose, indicating that there was limited cellulose depolymerization in ripe peach mesocarp tissue (Figure 1B). Brummell et al. [6] found that matrix glycan depolymerization is a controlled process, resulting predominantly from cleavage near the regions attached to cellulose, rather than wholesale cleavage in the regions spanning between microfibrils. These results suggest that depolymerization of cellulose is not a major factor in peach softening. Therefore, the changes in firmness during ripening are associated with pectin solubilization and are an alternative model that differs from the development stage.

### 4.3. Downregulation of PpCTL1 Suppresses Fruit Firmness by Interfering with Cellulose Content

It is well established that cellulose is synthesized by the cellulose synthase complexes (CSCs) formed by multiple isoforms of CesA enzymes [10]. In addition, other proteins play an important role in cellulose biosynthesis, such as chitinase-like proteins. In *Arabidopsis*, mutations in the *AtCTL1* or *AtCTL2* change the crystalline cellulose content in the cell wall [32,33,35]. Furthermore, CTL orthologs have been shown to be essential for cell wall structure and cellulose synthesis in a variety of plants [32,43,44]. In this study, we identified PpCTL1 and PpCTL2 with a conserved domain of Glyco_hydo_19 in peaches, which clustered with AtCTL1/2 in the CTL phylogenetic group (Figure 2), suggesting that these homologous genes may play similar biological roles. The transcript of *PpCTL1* was ubiquitously expressed in all tissues tested except roots (Figure 3A). On the contrary, the accumulation of *PpCTL2* expression was restricted to stems. Although, the expression levels of *PpCTL1/2* were reduced during fruit development and softening, consistent with a reduction in cellulose, the transcriptional accumulation of *PpCTL2* in fruit was very low, indicating that *PpCTL2* probably has limited effect on cellulose synthesis in peaches (Figure 3B,C).

To further elucidate the function of *PpCTL1* in peaches, the VIGS technique was used to inhibit *PpCTL1* expression in fruit using RNAi vectors (Figure 4). The results showed that the firmness of RNAi fruit was significantly lower than that of control fruit at 5 days after injection (DAI), coinciding with the downregulation of *PpCTL1* expression. Moreover, the RNAi fruit exhibited reduced cellulose content compared to control fruit, while there were no significant differences in acid-soluble pectin (ASP), water-soluble pectin (WSP) (expected at 10 DAI) content or cell wall structure (Figure 5). These results suggest that the downregulation of *PpCTL1* expression may lead to decreased cellulose content, consequently negatively impacting fruit firmness. Similar results were observed in studies on rice, where a mutation in the *BC15/OsCTL1* gene resulted in reduced cellulose content and weakened mechanical strength, leading to easily breakable internodes and leaves [45]. In maize, overexpression of *ZmCtl1* enhanced the mechanical stalk strength of the stem, supporting a role for *ZmCtl1* in tensile strength enhancement [13]. In conjunction with our study, these findings suggest a conserved role of CTL in regulating cellulose biosynthesis across different plant species. To our best knowledge, this is the first report highlighting the function of CTL in fruit.

The functional mechanism of chitinase in plants remains unclear because the substrate of chitinase, chitin, is lacking in plants [23]. At present, it is speculated that the substrate of chitinase may be arabinogalactan protein (AGP), which is important for plant growth and development as a glycoprotein on the cell wall [46,47]. Chitinase-like proteins isolated from carrot embryogenic cell lines have been shown to cleave AGPs, suggesting that AGPs might serve as the endogenous substrates for these chitinase-like proteins [46]. However, due to the absence of the chitinase activity-conserved H-E-T-T motif, *PpCTL1* may possess chitin binding activity but lack hydrolytic activity, thereby failing to participate in plant defense (Figure 2). Similarly, *AtCTL1* (homologous to *PpCTL1*) has shown no detectable chitinase activity [35]. Further studies suggest that *AtCTL1* can bind glucan polymers in vitro, indicating that *AtCTL1* may acts as a protein or carbohydrate scaffold to facilitate the interaction between glucan fibers, thereby influencing the cellulose–hemicellulose network [35]. Moreover, further studies are needed to elucidate the precise impact of *PpCTL1* on cellulose biosynthesis.

## 5. Conclusions

During fruit development, the decrease in firmness was associated with changes in cellulose content, whereas during ripening, fruit softening was related to the conversion of ASP (insoluble pectin) to WSP (soluble pectin). In addition, we identified two CTL genes, *PpCTL1* and *PpCTL2*, in peaches, exhibiting distinct tissue-specific expression patterns. During fruit development, VIGS silencing of *PpCTL1* resulted in reduced fruit firmness and cellulose content, while cell structure and ASP content remained unaffected. Taken together, our results suggest that *PpCTL1* plays an important role in maintaining fruit firmness by influencing cellulose biosynthesis.

The data provided evidence for the role of chitinase-like protein in peach development by describing changes in cell wall components. These findings lay the groundwork for further research into how *PpCTL1* can improve peach fruit texture and enhance our understanding of the molecular basis of fruit quality. Our future work will focus on understanding the precise impact of *PpCTL1* on cellulose biosynthesis. 

## Figures and Tables

**Table 1:** Correlation between fruit firmness with WSP, ASP and cellulose during development and softening stage.

	Firmness	
	Development Stage	Softening Stage
Firmness	1	1
WSP	0.595	−0.937 *
ASP	0.733	0.998 **
Cellulose	0.970 *	0.737

* and ** indicate significant linear correlation at *p* < 0.05 and 0.01 levels, respectively. Correlation analysis was performed by SPSS software 23.0. WSP, water-soluble pectin; ASP, acid-soluble pectin.

## Data Availability

Data is contained within the article or Supplementary Materials.

## Supplementary Materials

The following supporting information can be downloaded at: https://www.mdpi.com/article/10.3390/foods12132503/s1, Figure S1 Peach fruit growth curve. The fruit weight variations are shown for peaches at different development. S1 (20 DAB, days after blooming), S2 (35 DAB), S3 (50 DAB), S4 (65 DAB) and softening (75 DAB). Data are expressed as means ± SD from at least 15 fruits. Figure S2. Vector construction of TRV-PpCTL1. Figure S3. Multiple sequence alignment of PpCTL1/2 and AtCTL1/2 proteins. Different colors indicate different similarities (black: 100%, magenta: 75% and blue: 50%). Table S1. The information of the primers.
