# Supplementary material for: Chitinase-Like Protein PpCTL1 Contributes to Maintaining Fruit Firmness by Affecting Cellulose Biosynthesis during Peach Development
Source: Foods. 2023 Jun 27;12(13):2503. doi: 10.3390/foods12132503 (PMC10340415; doi:10.3390/foods12132503)
Supplement: Supplementary file 1 [file foods-12-02503-s001.zip › foods-2439933-supplementary.pdf]

Supplementary Material

Figure S1. Peach fruit growth curve.

Figure S2. Vector construction of TRV-PpCTL1.

Figure S3. Multiple sequence alignment of PpCTL1/2 and AtCTL1/2 proteins. Different color indicates different similarities (black: 100%, magenta: 75%, blue: 50%).

Table S1. The information of the primers.

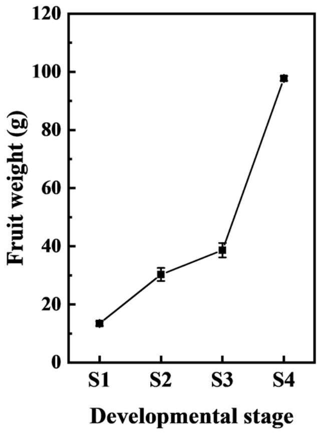

Figure S1. Peach fruit growth curve. The fruit weight variations are shown for peach fruit at different development. S1 (20 DAB, days after blooming), S2 (35 DAB), S3 (50 DAB), S4 (65 DAB), softening (75 DAB). Data are expressed as means ±SD from at least 15 fruit.

```
TRV2-PpCTL1  ..........................................................................................  0
PpCTL1       ATGAAGATTTCCACTGCTGCATTGGCGGTGGCGGTGGTGCTGAGTGTTTTCTTAGGTGCCAGCAGCGACGGCGATATGCCGTCAACGACC  90

TRV2-PpCTL1  ..........................................................................................  0
PpCTL1       CCGCGGGTGAAGACGGTGAGGGGGAAGAAGGTGTGTGACAGAGGGTGGGAGTGTAAAGGGTGGTCCCAGTACTGTTGCAATCTCACCATC  180

TRV2-PpCTL1  ..........................................................................................  0
PpCTL1       TCTGATTTCTTCCAAACTTACCAGTTTGAGAATCTCTTCTCCAAAAGAAACACGCCGGTGGCGCATGCCGTTGGGTTTTGGGATTATCAG  270

TRV2-PpCTL1  ..........................................................................................  0
PpCTL1       TCGTTTATTACTGCTTCTGCCCTCTTCCAGCCTCTTGGGTTCGGGACCACTGGTGGGAAGCTTATGCAGATGAAGGAGATCGCCGCCTTT  360

TRV2-PpCTL1  ..........................................................................................  0
PpCTL1       CTTGGACATGTCGGCAGCAAAACCACTTGTGGTTATGGTGTGGCCACAGGAGGACCATTGGCCTGGGGACTTTGCTACAACAGGGAAATG  450

TRV2-PpCTL1  ..........................................................................................  0
PpCTL1       AGTCCCATGCAGTCATACTGCGATGACTACTACAAATACACATACCCCTGCACTCCTGGAGCTGAATACTATGGTCGTGGTGCTTTGCCA  540

TRV2-PpCTL1  ................................................................CCGAATACATTGAGCAGAATGCTACT  26
PpCTL1       ATCTACTGGAACTACAATTATGGTGCAGCTGGAGAAGCTTTGAAGGTTGATCTGTTGAACCATCCCGAATACATTGAGCAGAATGCTACT  630

TRV2-PpCTL1  CTTGCTTTCCAGGCTGCAATATGGAGGTGGATGACTGCTATCAAGAAGTCACAACCCTCAGCGCACGATGCATTTGTTGGCAATTGGAAG  116
PpCTL1       CTTGCTTTCCAGGCTGCAATATGGAGGTGGATGACTGCTATCAAGAAGTCACAACCCTCAGCGCACGATGCATTTGTTGGCAATTGGAAG  720

TRV2-PpCTL1  CCCACCAAGAATGATACTTTGAGCAAGAGGTTTCCTGGATTTGGTGCTACAATGAATATTCTTTATGGCGATCAACTTTGTGGGCAGGGC  206
PpCTL1       CCCACCAAGAATGATACTTTGAGCAAGAGGTTTCCTGGATTTGGTGCTACAATGAATATTCTTTATGGCGATCAACTTTGTGGGCAGGGC  810

TRV2-PpCTL1  GACATTGATGCCATGAACACCATCGTTTCCCATTACCAGTACTACCTTGACCTTATGGGTGTCGGACGAGAGGAAGCAGGGCCCCATGAA  296
PpCTL1       GACATTGATGCCATGAACACCATCGTTTCCCATTACCAGTACTACCTTGACCTTATGGGTGTCGGACGAGAGGAAGCAGGGCCCCATGAA  900

TRV2-PpCTL1  GTGCTAACTTGTGCCGAGCAGGTTGCATTTAACCCAACCAAGGCTGCTGTTAC............                       349
PpCTL1       GTGCTAACTTGTGCCGAGCAGGTTGCATTTAACCCAACCAAGGCTGCTGTTACTGCATCTTCTTG                       965
```

Figure S2. Vector construction of TRV-PpCTL1.

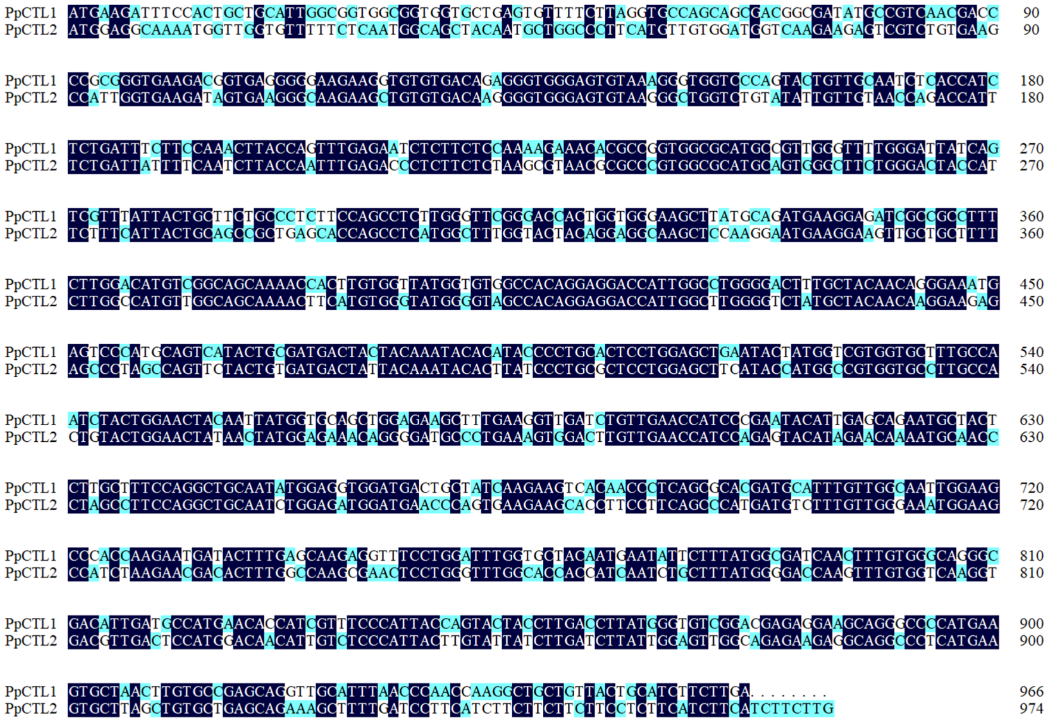

Figure S3. Multiple sequence alignment of PpCTL1/2 and AtCTL1/2 proteins. Different color indicates different similarities (black: 100%, magenta: 75%, blue: 50%).

Table S1. The information of the primers.

Symbol	Purpose	Gene number	Primer sequence(5'-3')(Forwad/Reverse)
PpCTL1	Cloning	ppa008791m	GCTGAATACTATGGTCGTGGTGCT/ CGCTGAGGGTTGTGACTTCTTGAT
PpCTL2	Cloning	ppa000305m	GGAAGCCATCTAAGAACGACACT/ CCTCTTCTCTGCCAACTCCAATA
v-PpCTL1	Vector construction	ppa006392m	CGGAATTCCCGAATACATTGAGCAGAAT/ GGGGTACCGTAACAGCAGCCTTGGTT
